# Supplementary figures and images for: Evolution of gremlin 2 in cetartiodactyl mammals: gene loss coincides with lack of upper jaw incisors in ruminants
Source: PeerJ. 2017 Jan 26;5:e2901. doi: 10.7717/peerj.2901 (PMC5274524; doi:10.7717/peerj.2901)

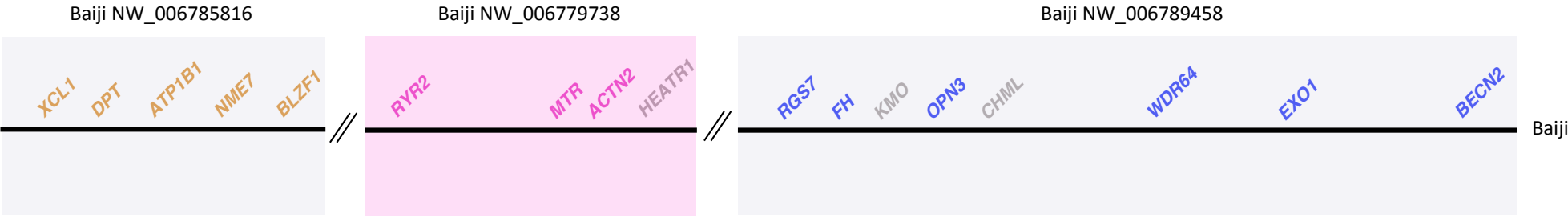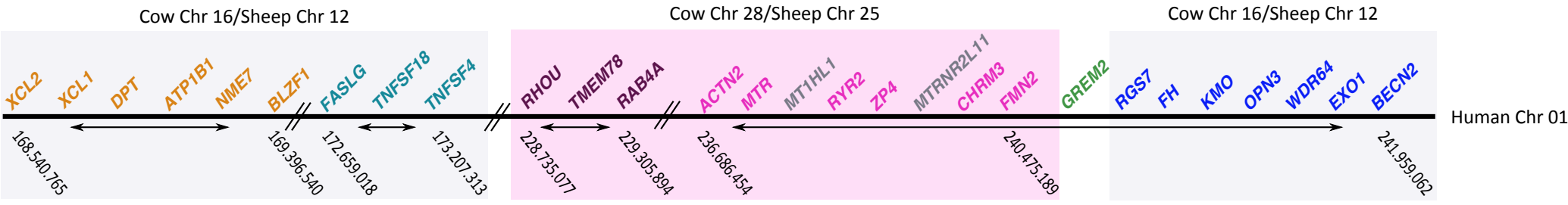

Supplement: Supplemental Information 1 [file peerj-05-2901-s001.zip › Supplementary_Fig_1.pdf]
